# TRPV1 and Endocannabinoids: Emerging Molecular Signals that Modulate Mammalian Vision

**DOI:** 10.3390/cells3030914

**Published:** 2014-09-12

**Authors:** Daniel A. Ryskamp, Sarah Redmon, Andrew O. Jo, David Križaj

**Affiliations:** 1 Department of Ophthalmology & Visual Sciences, Moran Eye Institute, University of Utah School of Medicine, Salt Lake City, UT 84132, USA; E-Mails: sarah.redmon@utah.edu (S.R.); u0751604@utah.edu (A.O.J.); 2 Interdepartmental Program in Neuroscience, University of Utah School of Medicine, Salt Lake City, UT 84132, USA; 3 Department of Neurobiology & Anatomy, University of Utah School of Medicine, Salt Lake City, UT 84132, USA; 4 Center for Translational Medicine, University of Utah School of Medicine, Salt Lake City, UT 84132, USA

**Keywords:** retinal ganglion cells, TRPV1 channels, endocannabinoids, CB1 receptors

## Abstract

Transient Receptor Potential Vanilloid 1 (TRPV1) subunits form a polymodal cation channel responsive to capsaicin, heat, acidity and endogenous metabolites of polyunsaturated fatty acids. While originally reported to serve as a pain and heat detector in the peripheral nervous system, TRPV1 has been implicated in the modulation of blood flow and osmoregulation but also neurotransmission, postsynaptic neuronal excitability and synaptic plasticity within the central nervous system. In addition to its central role in nociception, evidence is accumulating that TRPV1 contributes to stimulus transduction and/or processing in other sensory modalities, including thermosensation, mechanotransduction and vision. For example, TRPV1, in conjunction with intrinsic cannabinoid signaling, might contribute to retinal ganglion cell (RGC) axonal transport and excitability, cytokine release from microglial cells and regulation of retinal vasculature. While excessive TRPV1 activity was proposed to induce RGC excitotoxicity, physiological TRPV1 activity might serve a neuroprotective function within the complex context of retinal endocannabinoid signaling. In this review we evaluate the current evidence for localization and function of TRPV1 channels within the mammalian retina and explore the potential interaction of this intriguing nociceptor with endogenous agonists and modulators.

## 1. Introduction

Transient Receptor Potential (TRP) channels form a superfamily of non-selective cation channels that provide cells and organs within the vertebrate body with information about the external and internal environment. The channels participate in the sensory transduction of light, pain, touch, temperature, osmolarity, taste, pheromones, acidity, inflammation, oxidation, metabolic energy and polyunsaturated fatty acids [1,2,3,4,5,6,7], whereas loss- and gain-of-function TRP channel mutations cause diseases that range from visceral organ failure (TRPP2/polycystic kidney disease; TRPC6/focal and segmental glomerulosclerosis) to skeletomuscular dysplasias (TRPV4/Charcot-Marie-Tooth disease type 2C), locomotor dysfunction (TRPC3; cerebellar ataxia) and blindness (TRPM1; congenital stationary night blindness) [8]. Within the brain, TRP channels have increasingly been shown to play essential and supportive roles in neuronal and glial signaling [9,10] through monitoring of systemic osmo- and thermoregulation [11,12], neuro-glial-vascular coupling [13,14], initiation of neural stress responses [15], neuroprotection [16] and neurodegeneration [17]. Despite the accumulated knowledge, the primary functions for most TRP isoforms expressed in a cell type-specific manner across the vertebrate central nervous system (CNS) remain poorly understood. Functional information on TRP signaling is particularly scarce for the retina, which, however, expresses the transcripts of most, if not all, known TRP channel isoforms [18]. Localization of TRPs in the retina has been difficult given the non-specificity of most available antibodies [18,19,20]; however, evidence suggests that expression of some channels (e.g., TRPM1 & 3, TRPC6, TRPC7 and TRPV4) may be confined to specific subsets of retinal cells [10,17,21,22,23], whereas others (e.g., TRPC1, TRPC3, TRPML1, TRPM7 and TRPP2) appear to be expressed across multiple retinal layers [18,19]. The absence of functional information on TRP signaling in vertebrate retinas appears ironic given that the original dTRP channel was discovered as a light-activated photochannel in *Drosophila* photoreceptors [1,24]. Nonetheless, amongst the important discoveries from recent years is the identification of TRPM1 as the mGluR6-gated cation channel that is required for transmission of the light signal in ON bipolar cells [10,25], discovery that the *Trpm3* gene and its intronically hosted micro-RNA gene (miR-204) are localized to cells residing within the inner nuclear layer [18,26] and the demonstration of the central role of TRPC6/7 heteromers in phototransduction by melanopsin-expressing RGCs [22]. These studies showed that TRP isoforms play fundamental and irreplaceable functions in vertebrate vision. Here, we review potential roles for the vanilloid isoform 1 (TRPV1), which while arguably one of the most thoroughly studied TRP channels within the PNS and CNS, has remained understudied within the context of retinal physiology and visual signaling.

TRPV1 was first identified by Julius and coworkers when a single clone of cDNA conferred responsiveness to the spicy ingredient from hot chili peppers, capsaicin [2]. With six transmembrane domains, a pore between segments 5 and 6 and large intracellular N- and C-termini, this tetrameric ligand-gated channel shares a common structure with the other 27 mammalian TRPs as well as with the voltage-activated potassium (K_v_) channel family [27]. The 6 ankyrin repeats within the N-terminus are likely to mediate protein-protein, protein-cytoskeleton and heteromeric interactions as well as trafficking, ligand binding and modulation by ATP and calmodulin [7,28,29,30]. The channel, at −60 mV, conducts a slowly developing nonselective cation current with a P_Ca_/P_Na_ of 9.6 and a single channel conductance of ~−80 pS at positive and −40 pS at negative membrane potentials. TRPV1 desensitizes in response to calmodulin binding to N- and C-termini but may change its cation permeability during prolonged agonist stimulation, following exposure to protons and/or phosphorylation [2,31,32]. Indeed, Ca^2+^ entry through TRPV1 can be large enough to cause a self-impacting negative feedback on channel permeability and downregulation of voltage-operated Ca^2+^ channels [33]. The responses of the channel are additionally determined by splice variations [12], heteromultimerization with other TRP channel subunits including TRPV2-4 [34], phosphatidylinositol 4,5-biphosphate (PIP2) and other membrane-delimited lipids [35,36,37] and insertion/internalization [38,39]. A pivotal role for TRPV1, validated by genetic ablation, small interfering RNA knockdown and pharmacological experiments [15,40] is dynamic modulation of the neuronal response to injury that leads to nociception and hyperalgesia. The channel also contributes to pain transduction through polymodal integration of stimuli such as chemicals (capsaicin, resiniferatoxin or gingerol), pain, temperature (>42 °C), acidity, shrinking, endocannabinoids (eCBs) and eicosanoids [2,36,41,42].

TRPV1 permeability and gating are fine-tuned by a multitude of direct and indirect mechanisms. The channel is indirectly sensitized by inflammatory mediators such as bradykinin, leukotriene B4, histamines and prostaglandins that impact it in part through heteromeric G proteins and tyrosine kinase pathways, whereas certain stimuli (heat, protons, voltage) sensitize the channel to the other agonists [43,44]. TRPV1 also contains consensus sites for protein kinases A and C and src tyrosine kinases that regulate its inactivation properties through phosphorylation [45,46,47,48] and should therefore be viewed as a complex, highly modulatable sensory switch which can be flipped on or off by combinatorial action of modulators, agonists and the intra-/extracellular context [42,43,49]. Although functional data on TRPV1 obtained for any particular tissue, cell type, or condition are not automatically generalizable, it may be helpful to consider studies of TRPV1 in the brain to appreciate the state of our understanding of TRPV1 in its visual extension, the retina.

## 2. TRPV1 in the Brain

### 2.1. TRPV1 Expression in the Brain

Although TRPV1 expression is ~20-30-fold lower in the CNS compared to the dorsal root ganglion [50,51,52], a combination of pharmacological, genetic and biochemical data suggest widespread distribution across the neural axis from the spinal cord to brain areas that may include the hippocampus, hypothalamus, thalamus, cerebellum, cortex and limbic system [53,54,55]. Accordingly, functional studies have implicated the channel in drug addiction, anxiety/fear behavior and long-term memory formation [56,57,58]. Injection of capsaicin induced changes in thermogenic behavior [59] and locomotion [60], presumably by acting on hypothalamic and striatal circuits. Because antibodies, radioligand binding and reverse transcription (RT)-PCR assays have provided highly variable localization evidence for TRPV1 expression across the brain, and because studies that used similar assays often reached different conclusions regarding the contribution of the channel to neuronal function [20,30,61,62,63], there seems to be no uniform consensus on the distribution of TRPV1 channels in the CNS. Analysis of TRPV1 distribution is further complicated by the heterogeneous expression of TRPV1 splice variants, compensatory effects in KO mice, differences in age-dependent phenotypes/expression and differential expression across mouse strains/backgrounds [12,50]. Functional studies have led to variable conclusions regarding the role of the channel in synaptic plasticity [56,57,64]. A paradigmatic example that challenges much of the previous work is the recent analysis of Trpv1^PLAP–nlacZ^ reporter mice which suggests that TRPV1 expression within the brain is highly restricted, with the most pronounced reporter signals observed within the caudal hypothalamus [51], which shares with the retina the susceptibility to high doses of systemic capsaicin [65,66]. It is possible, however, that use of reporter mice might have missed alternate TRPV1 isoforms and/or resulted in an artificial decrease in endogenous TRPV1 levels [55]. Thus, although many details with respect to the cellular/regional distribution of TRPV1 remain to be resolved, it is likely that the channel has a broader distribution and range of functions within the CNS than generally thought.

## 3. TRPV1 in the Retina

### 3.1. Overview of Retinal Anatomy and Early Visual Information Processing

**Figure 1:**
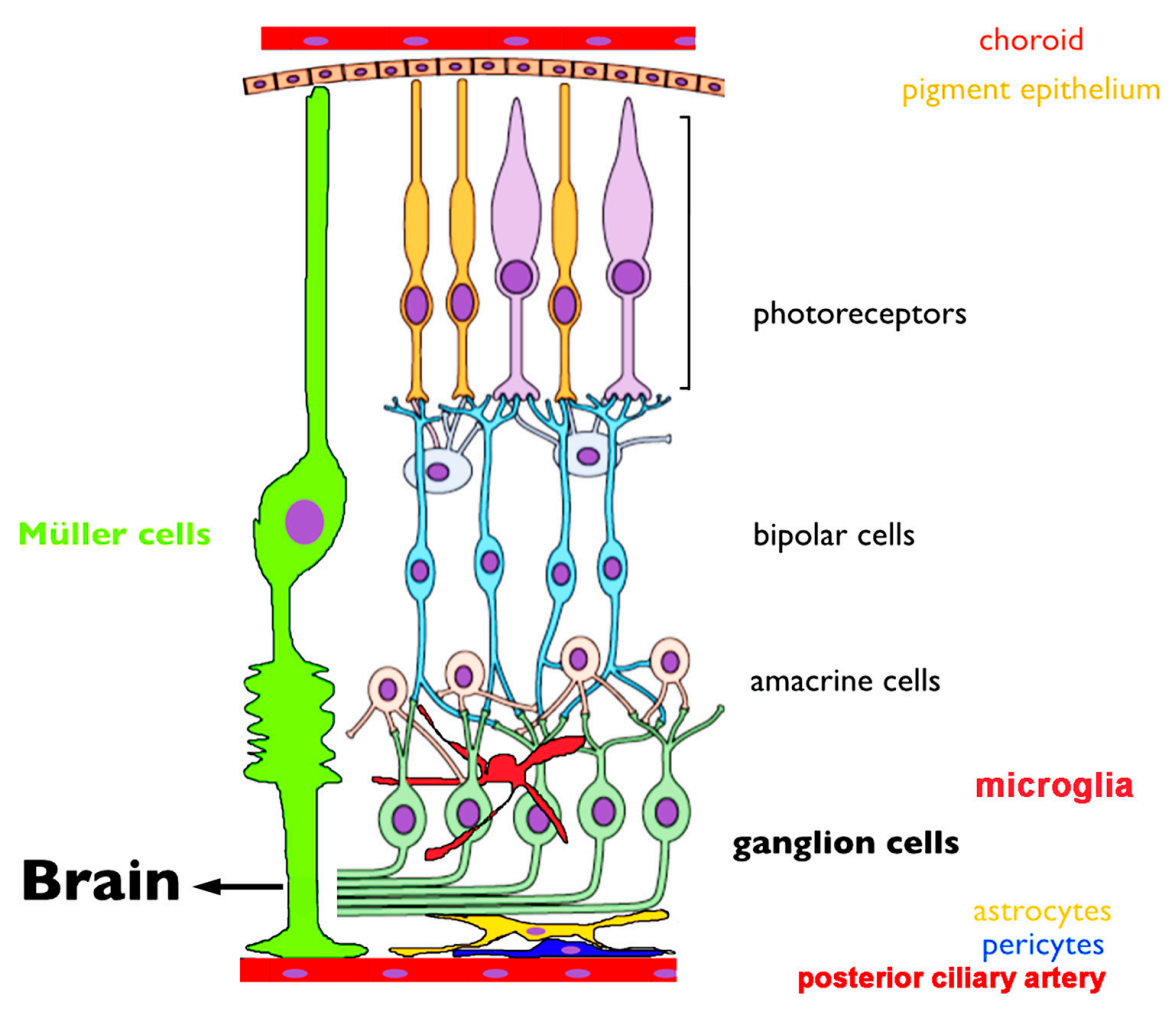
A schematic of the retina showing overall arrangement of retinal layers and relationship to the critical vascular and pigment layers. An excitatory vertical chain (photoreceptors, bipolar cells, ganglion cells) provides a direct route for transmitting visual information to the midbrain. Lateral inputs from horizontal cells and amacrine cells provide luminance gain control and organization spatiotemporal receptive fields of retinal neurons. Müller glia provide most of the functions performed by astrocytes in the brain. Astrocytes form the blood-retina barrier together with pericytes and vascular endothelial cells. Microglia play a role in developmental pruning and the retinal immune response whereas the choroid and posterior ciliary artery feed outer and inner retinal neurons, respectively. Adapted from [70].

When light enters the eye, it is focused onto the retina by the lens and transduced by photoreceptors into electrical and chemical signals that are processed as they are passed through retinal circuits towards the brain. In a nutshell, the flow of visual signal consists of a vertical glutamatergic signals mediated by excitatory photoreceptor, bipolar and ganglion cell synapses and inhibitory/modulatory contributions from horizontal networks mediated by horizontal and amacrine cells, respectively (Figure 1).

Absorption of photons results in the reduction of tonic release of glutamate to postsynaptic horizontal cells and bipolar cells, respectively. Bipolar cells transmit this information to RGCs through two opposing yet complementary pathways. A non-inverting synapse to OFF bipolar cells mediates the excitatory response to decrements of light through ionotropic AMPA/KA receptors. Conversely, the ON pathway is based on a metabotropic mGluR6- TRPM1 mechanism whereby glutamate tonically suppresses bipolar cells and thus cells become disinhibited by light-induced suppression of glutamate release from rods and cones. As bipolar cells forward feed visual information, it is processed through parallel and serial pathways that involve feedback interactions with amacrine cells that contribute to lateral inhibition that sculpts the spatiotemporal organization of RGC receptive fields. As shown in Figure 1, RGCs represent the final output pathway that conveys the visual signal to the midbrain [67]. RGC function and output can be influenced by retinal glia that include Müller cells that span the tissue (and provide critical ionic, metabolic and modulatory of support for neurons, [68]), protoplasmic astrocytes that line the inner limiting membrane (ILM) and (in vascularized retinas) control the permeability of the blood-retina barrier, and microglia, which represent the resident immune cells [69]. Whilst organization of retinal circuits represents a key determinant of visual information processing, the physiological state of every cell type is dynamically altered through activity-dependent and neurodegeneration-driven changes in calcium homeostasis, functional and structural connectivity across and between retinal laminae [67,69,70]. TRP channels represent new players that play increasingly visible roles in retinal neuronal and glial Ca homeostasis by transducing the RGC response to light and driving plasma membrane Ca influx as well as through interactions with intracellular Ca stores, heteromerization and activation of purinergic receptors and pannexin hemichannels [17,18,19,21,22,25,71,72,73,74,75].

### 3.2. TRPV1 Channel Distribution in the Retina

The first evidence suggestive of TRPV1 expression in the retina was obtained by Ritter and Dinh [66,76], years before the seminal cloning of TRPV1 in dorsal root ganglia (DRG) [2]. The investigators used cupric silver staining to reveal capsaicin-induced neurodegeneration in the rat retina. This effect was observed in the IPL and RGCL and was also associated with impaired anterograde axonal transport [77]. Interestingly, unilateral transection of the optic nerve prior to capsaicin administration prevented contralateral spread of neurodegeneration to RGC projection sites within the midbrain [66]. In some cases, capsaicin treatment appeared to damage RGC projections without destroying the soma [76], consistent with the documented ability of capsaicin to act on axons in peripheral nerves [78]. Subsequently, Szallasi *et al.* [61], used autoradiography of whole-head cryosections to confirm the retina as a binding site for the TRPV1 agonists capsaicin and its ultrapotent analog, the *Euphorbia resinifera*-derived resiniferatoxin.

Some of these early studies may need to be re-evaluated. Similar to the limitations of approaches that exclusively rely on antibody staining, results from studies confined to the use of pharmacological agents such as capsaicin (agonist) and capsazepine (antagonist) should take into account non-specific and/or mis-targeting effects. TRPV1 was originally called the ‘capsaicin receptor’ based on the active ingredient in *Capsaicum* chili peppers; however, capsaicin has been subsequently shown to also affect a range of TRPV1-independent mechanisms that include activation of voltage-gated inward and outward currents, impairment of mitochondrial function, inhibition of prostaglandin E2 production and importantly, binding to the cannabinoid CB1 receptor [79,80,81,82]. The use of the agonist is particularly complex within the retinal context, as capsaicin induces large inward currents in ON-bipolar cells that mediate an excitatory, inverted, photoreceptor response towards the inner retina. Capsaicin-evoked responses within rod and cone ON bipolar cells at first appeared to be indistinguishable from the effects mediated by light and mGluR6 antagonists and therefore pointed at a “TRPV1-like” identity of the long-sought transduction channel. However, it was soon discovered that ON bipolar light responses are unaffected in Trpv1^−/−^ mice, whereas they are eliminated by deletion of another TRP channel, the melastatin receptor TRPM1 [10]. While this suggested that capsaicin sensitivity is mediated through TRPM1, only partial reduction in the amplitude of capsaicin responses was observed in Trpm1^−/−^ mice [25], indicating that TRPM1 might not represent the exclusive capsaicin sensor in ON bipolars. Consistent with this, Xu *et al.* [83] recently reported that the capsaicin response is eliminated in mice lacking the mGluR6 receptor, suggesting that the compound also acts on the metabotropic glutamate receptor or its downstream G_o_ protein heteromers. Irrespective of the precise mechanism, it is especially important to consider potential non-TRPV1-mediated effects when capsaicin is used to analyze retinal function or pathology. The results obtained by using the popular competitive antagonist capsazepine should likewise be interpreted with caution, as the drug can produce effects in Trpv1^−/−^ mice. These include antagonism of voltage-activated Ca^2+^ channels [84], acetylcholine receptors [85], hyperpolarization-activated cation channels (I_h_) [86] and stimulation of amiloride-sensitive ENaC channels [87].

Nonetheless, numerous studies demonstrated the presence of TRPV1 mRNA and protein in the mammalian retina as well as in teleosts, amphibians and avians [10,18,71,72,88,89], leading Zimov and Yazulla to suggest that the role of the channel in visual signaling might be conserved across vertebrates [90,91]. A recent study, using probes and antibodies that detected TRPV1 signals in DRG, suggested that TRPV1 mRNA and protein levels in the mouse retina are unusually low [18]. The differences between studies are likely to reflect use of different antibodies and staining protocols, but also underscore the need for using Trpv1^−/−^ mice as controls for semi-quantitative expression studies.

A potential role for TRPV1 in ontogeny was suggested by Leonelli *et al.* [92], who detected stable TRPV1 protein expression in embryonic and early postnatal (E19 through P14) rat retinas, followed by a marked increase in expression during the transition from eye opening to adulthood (at ~P60). Exposure of neonatal rats to capsazepine increased the prevalence of subsets of parvalbumin^+^ amacrine-like cells and increased immunoreactivity (ir) for the presynaptic amacrine marker synapsin 1b. Capsazepine also reduced the normal, developmentally driven, apoptosis in the isolated neonatal rat retina but did not affect retinal layering in the adults; thus, it was proposed that TRPV1 plays a role in the wiring of retinal circuits [93].

Similar to studies in the brain and non-excitable tissues [51,63], immunohistochemical (IHC) staining of mammalian retinas has yielded variable, antibody-specific patterns. The most commonly reported observation from studies that rely mainly on IHC is prominent TRPV1-ir in RGCs and astrocytes. In rat, TRPV1 labeling was confined to a subset of cells in the GCL and within the optic nerve head, but was weak within the optic nerve itself [92]. Further consistent with RGC localization, axotomy of the optic nerve resulted in decreased TRPV1 expression that was mirrored by an injury-induced decline in the population of TRPV1-expressing cells [88,94] and TRPV1-r was also detected within retinocollicular projections within the superior colliculus [95]. Suggestive of potential presynaptic function, TRPV1-ir puncta in the inner plexiform layer (IPL) colocalized with the synaptic vesicle marker synaptophysin. Increased density of labeled axon bundles near the optic nerve head was also observed together with the labeling of distinct cell types in the GCL that included large SMI32^+^ cell bodies, axons and dendrites and smaller Thy-1.1^+^ cells. Consistent with the labeling in retinal sections, TRPV1-ir was also observed in somata/dendrites/neurites of immunopanned and cultured RGCs [72]. Sappington *et al.* [72] reported that retinal TRPV1 expression increases with intraocular pressure (IOP) and suggested that mechanical activation of the channel induced by chronic increases in IOP may drive progressive degeneration of RGCs.

T*rpv1* mRNA signals in immunomagnetically separated postnatal and adult rat RGCs are weaker compared to the whole retina [72], indicating the transcripts are likely to be generated in other cell types in addition to RGCs. Accordingly, immunolocalization and pharmacological assays implicated photoreceptors, bipolar cells, amacrine cells and glia as potential TRPV1 expressors in rodents, teleosts and primates [92,96,97,98]. TRPV1-ir was observed in synaptic terminals of salamander and rat photoreceptors [92,98,99], suggesting that the channel could modulate the transmission of the response to postsynaptic bipolar and horizontal cells. However, arguing against a major role in phototransduction and neurotransmission in the outer retina, photopic and scotopic ERG a- and b- waves in Trpv1^−/−^ mice are indistinguishable from field potentials in wild type animals [10]. It is possible, however, that the diffuse TRPV1-ir observed in the OPL [72,100] corresponds to Müller glial processes and/or resident microglia. Consistent with the microglial locus, TRPV1-ir is present in Iba1^+^ and OX-42^+^ cells whereas cultured retinal microglia respond to capsaicin [71,92]. TRPV1 expression in Müller cells is less clear. Leonelli *et al.* [92] did not detect TRPV1-ir in rat Müller glia whereas Martinez-Garcia *et al.* [89] reported TRPV1-ir in rabbit Müller cells. Thus, localization to photoreceptors, Müller glia, bipolar cells and/or amacrine cells may be species-specific and the identity of TRPV1 signals in the mammalian retina remains a subject for further studies. Leonelli *et al.* [92] detected weak TRPV1-ir puncta in astrocytes that line the vasculature at the inner limiting membrane (Figure 1). This was recently confirmed in the functional study by Ho *et al.* [101] who provided evidence that the channel plays a role in guiding astrocyte migration following wound injury. Finally, TRPV1-ir was also reported in the retinal pigment epithelium in rabbits and humans [89], indicating a yet-to-be-defined role in the ion transport function of these cells that are critically important for photoreceptor function and survival.

**Figure 2:**
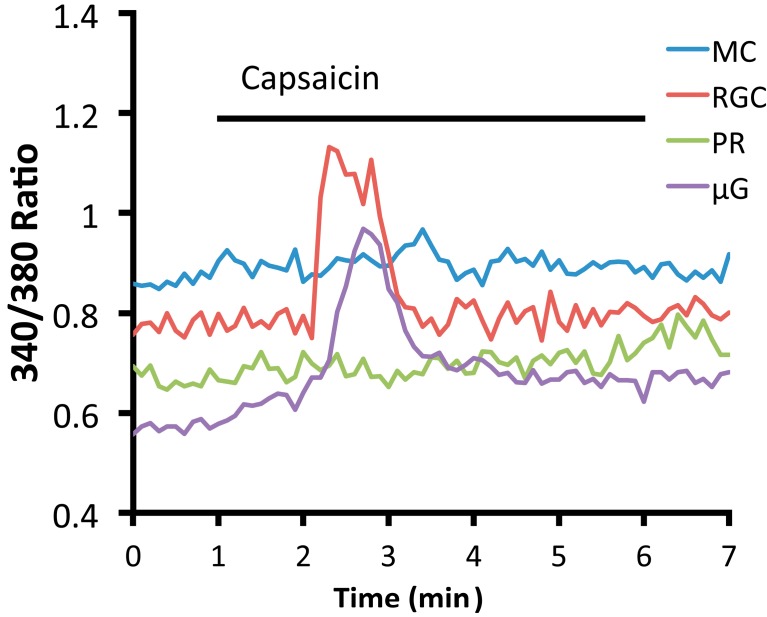
An acutely isolated and dissociated mouse RGC (blue) and microglial cell (µG; green) loaded with fura-2 respond to capsaicin (40 µM) with transient [Ca^2+^]_i_ elevations that inactivate during stimulus application, whereas a representative photoreceptor (PR; purple) and Müller cell (MC; red) do not respond to the TRPV1 agonist (see [19,103] for methods).

The variable results obtained with TRPV1 antibodies and pharmacological agents suggest the need for additional functional assays as well as control experiments based on the widely available mice with the genetically ablated channel. One example of such an auxiliary method is optical imaging, which indeed shows robust, desensitizing, capsaicin-induced [Ca^2+^]_i_ elevations in mouse RGC somata and microglial cells, but not in Müller astroglia or photoreceptors (Figure 2). The identity of the channel was additionally confirmed by blocking capsaicin-evoked [Ca^2+^]_i_ elevations with capsazepine and by showing their absence in Trpv1^−/−^ cells [102]. Functional expression of TRPV1 in RGCs and microglial cells, together with their absence from photoreceptors and bipolar cells suggests that the channel may play a more significant function within the inner retina.

### 3.3. Endogenous and Synthetic TRPV1 Agonists and Retinal Signaling

Because TRPV1 receptors are unlikely to experience conspicuous nociceptive, mechanical and noxious thermal stimuli in the healthy retina, their primary activators of are likely to be endogenous polyunsaturated fatty acids. TRPV1 is gated by precursors and derivatives of arachidonic acid commonly lumped together into the “endovanilloid/endocannabinoid” (eCB) category of signaling metabolites that are ubiquitous across all retinal layers [98]. These may include anandamide (Arachidonoyl ethanolamide; AEA), unsaturated N-acyl-dopamines (e.g., N-Arachidonoyl dopamine; NADA), 2-arachidonoglycerol (2-AG, an ester formed from arachidonic acid and glycerol) and lipoxygenase metabolites of arachidonic acid (hydroperoxyeicosatetraenoic acids; HpETEs) [36] (Figure 2).

**Figure 3:**
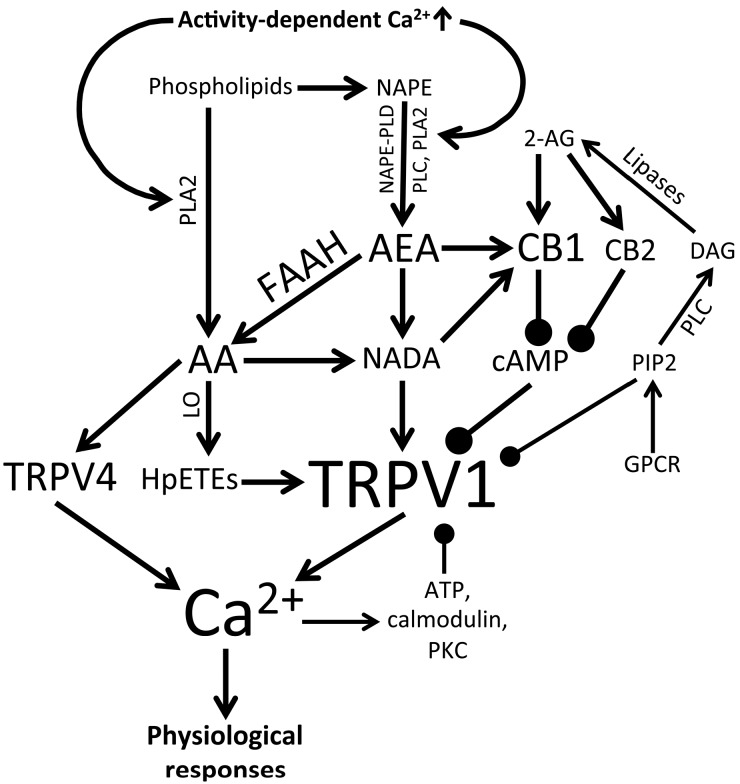
Cellular activity initiates synthesis of endocannabinoids/endovanilloids, which activate (arrows) or modulate (closed circles) TRPV1 and TRPV4 activity and thereby regulate physiological responses. 2-AG (2-arachidonoylglycerol), AA (arachidonic acid), AEA (anandamide), CB1 (cannabinoid receptor type I), CB2 (cannabinoid receptor type I), DAG (diacylglycerol), FAAH (fatty acid amide hydrolase), GPCR (G protein-coupled receptor), HpETEs (hydroperoxyeicosatetraenoic acids), LO (lipoxygenase), NADA (N-arachidonoyl dopamine), NAPE (N-acylphosphatidylethanolamine), NAPE-PLD (NAPE-specific phospholipase D), PIP2 (phosphatidylinositol 4,5-bisphosphate), PKC (protein kinase C), PLA2 (phospholipase A2), PLC (phospholipase C).

eCBs affect TRPV1 activity directly and indirectly via activation of cannabinoid receptors (CBRs), a 2-isoform family of G protein-coupled receptors with ~44% amino acid similarity that are associated with intracellular cAMP metabolism and downstream activation of the MAPK-ERK pathway. Type I CB receptors (CB1Rs) are most abundant GPCR within the CNS and all TRPV1-expressing primary sensory neurons express CB1R *in vitro* [104]. The complexity of eCB composition translates to their biological effects. Thus, AEA activates TRPV1 and CB1Rs, whereas 2-AG is a full agonist of CB1Rs and CB2Rs but does not affect TRPV1 [105]. eCB biosynthesis from phospholipid precursors is primarily activity-dependent and follows activation of Ca^2+^-dependent phospholipases (including phospholipases A2 and C). For example, 2-AG (which, unlike anandamide is expressed at high levels in the CNS) is primarily synthesized by phospholipase C and by diacylglycerol lipase (DAGL) acting on the second messenger diacylglycerol (DAG) [106] (Figure 3). Both AEA and 2-AG freely diffuse across the membrane but can be released into extracellular space to affect neural activity via presynaptic and/or postsynaptic binding sites. eCBs target pre- and/or postsynaptic TRPV1 channels and were reported to bind the cognate TRPV2-4 isoforms with moderate-to-high efficacy and potency (EC_50_ ~3.7 µM) [107]. The EC_50_ values for binding of cannabinoid compounds to CBs and TRPV1 are typically in the micromolar range, although some (such as NADA) can bind TRPV1 in the nanomolar range [82]. Thus, eCB release will exert a multimodal effect through separate CBR and vanilloid receptor mechanisms, but may also trigger reciprocal interactions between the two. Interestingly, coactivation of excitatory (TRPV1) and inhibitory (CB1R) eCB-dependent mechanisms can be concentration-dependent whereby CB1R-mediated effects predominate at low (nM) levels of AEA and TRPV1-mediated effects are more pronounced at higher (~µM) concentrations [104]. Alternatively, the two effectors of AEA might be activated sequentially, with stimulation of high-affinity CB1Rs preceding subsequent activation of TRPV1 [108]. Moreover, reciprocal interaction between the GPCR and the ion channel may involve CB1R-mediated sensitization or desensitization of TRPV1 through PKA/PKC-dependent regulation of its phosphorylation state [109]. With respect to the retina, CB1R transcripts were found in both plexiform layers [99,110,111], whereas DAG lipases were localized to OFF bipolar cells [112]. Given the prominent localization of TRPV1 in the inner retina, it is plausible that Ca^2+^-dependent eCB release, associated with periods of intense ON- and OFF RGC activation results in indirect (CBR-mediated) and direct TRPV1 modulation. As if this is not complicated enough, arachidonic acid and its downstream eicosanoid metabolites also activate TRPV4, a polymodal vanilloid receptor homolog that acts as a RGC osmosensor [17]. The properties of natural stimuli (light) that lead to endocannabinoid concentrations high enough for retinal TRPV1 and/or CB1R activation remain to be identified.

Enzymatic synthesis and degradation of endovanilloid/eCBs is a likely rate-limiting step in TRPV1 activation and inhibition. AEA is degraded by the fatty acid amide hydrolase (FAAH), resulting in the production of ethanolamine and arachidonic acid (AA), whereas 2-AG can be hydrolyzed by monoacylglycerol lipase (MAGL), α/β-hydrolase domain 6, 12 (ABHD6/ABHD12), and/or cyclooxygenase-2 (COX-2) [for retinal distributions see [112,113]. FAAH is ubiquitously expressed across the brain and the retina, with expression reported in subsets of photoreceptors, RGCs, dopaminergic and cholinergic amacrine cells and horizontal cells [98,112,114]. IHC studies in fish colocalized FAAH with CB1Rs and TRPV1 within presynaptic terminals of the IPL and OPL [90,91]. FAAH expression in photoreceptors is consistent with the observed AEA uptake [100] and localization of CB1 receptors [99]. The NMDA inhibitor MK801 reduced retinal FAAH activity [88], possibly by downregulating the Ca^2+^-dependent modulation of the enzyme. FAAH-mediated catabolism of anandamide may be suppressed by PEA, which is present rat, cat and monkey retinas, potentiating eCB signaling *in vivo* [115]. The augmentation of eCB signaling by inhibition of FAAH can produce effects that markedly differ from those elicited by CB1 agonists [116], consistent with synergistic action on TRP channels. Likewise, the breakdown of the FAAH metabolite arachidonic acid by 12-lipoxygenase (12-LO) and 15-lipoxygenase (15-LO) generates 12(S)-HpETE and 15(S)-HpETE, which also bind and activate TRPV1 [117,118]. It would be interesting to see whether the retinas of FAAH^−/−^ mice favor eCB signaling over that of endovanilloids and how this affects the responses of retinas to light/darkness.

To understand TRPV1 function in the retina one must differentiate the stimulatory effects of endogenous and synthetic cannabinoids on visual signaling and their protective effects in pathological paradigms. Systemic exposure to marijuana produces a dazzling variety of visual experiences that include increased photosensitivity [119], changes in color discrimination [120] and double/blurred vision [96,121], with a mechanism of action that is likely to involve both retinal and cerebral targets. The psychoactive ingredient in marijuana, Δ^9^-tetrahydrocannibinol (THC), mimics eCBs through its stimulation of CB1 receptors but does not activate TRPV1. Cannabinoid effects within the retina were also shown to be complex under experimentally tractable conditions. Amongst the observed effects are modulation of intracellular signaling in most retinal cell types including potassium and calcium currents in amphibian and fish photoreceptors [96,122], voltage-operated currents in rodent RGCs [123] and transmitter release from amacrine and bipolar processes in teleosts and mice [97,124].

Data from exogenous CB1 agonists, eCB measurements, and FAAH inhibition has suggested an important role for eCB signaling in neuroprotection. THC lowers IOP and protects the retina from excitotoxic damage [96,125,126]. Analogously, synthetic cannabinoids evinced remarkable protection for photoreceptors and bipolar cells in a rat model of retinitis pigmentosa [127] and were suggested to reduce retinal pigment epithelium (RPE) and RGC damage in models of ischemia, glaucoma, diabetic retinopathy, oxidative damage and/or traumatic ocular injury. Suggestive of lost protection, decreased levels of 2-AG have been reported in ocular tissues from glaucoma patients [128]. FAAH inhibition elevates AEA in young, but not old, rat retinas, an effect that was associated with CB1-dependent RGC neuroprotection following axotomy [129]. In a study of ischemic-reperfusion (I-R), Nucci *et al.* [88] used high IOP (~120 mmHg) to block blood flow within and induce RGC loss in the rat retina. The cytotoxic effect of I-R was antagonized by systemic FAAH inhibition or intravitreal injection of MetAEA, a stable analogue of anandamide. MetAEA-mediated neuroprotection was blocked by inhibition of either CB1 or TRPV1, indicating that both receptors might be involved in neuroprotective effects of anandamide. Other studies showed that cells exposed to excitotoxic conditions can be rescued with eCBs, possibly by suppressing excessive release of glutamate, depolarization-mediated Ca^2+^ overloads and/or excessive firing within the retina [123,124,130]. It is unclear if TRPV1 and CB1Rs regulate the same neuroprotective pathway [but see 102]. Moreover, it remains to be seen whether CB1 and/or TRPV1 inhibition or genetic deletion exacerbate RGC loss in I-R. While eCBs act via CB1Rs [98], the consequences of parallel activation of TRPV1 were not assessed. Thus, it is not known whether CB1R activation protects retinal neurons synergistically with TRPV1 and/or CB1Rs provide protection by antagonizing Ca^2+^ overloads mediated by TPRV1. In either case, CB1Rs and TRPV1 represent novel potential targets that might confer neuroprotection within the retina without causing excitotoxicity. A bottleneck in investigating these potentially ubiquitous signaling pathways in the retina has been that, in contrast to the effects of synthetic cannabinoids applied to (isolated, cultured) retinal cells, the biological functions of eCBs, TRPV1 and their interactions across retinal circuits remain almost entirely unknown.

### 3.4. Is TRPV1 Neurodegenerative or Neuroprotective?

The expression of TRPV1 in the IPL and GCL [66,72,92] implies a potential role for pathological enhancement of RGC excitability, post-synaptic voltage-operated Ca^2+^ influx and glutamate receptor-mediated currents that lead to cell injury via Ca^2+^ overload. Consistent with this, maximal TRPV1 activation has been shown to cause neuronal and glial excitotoxicity in other areas of the brain [131,132,133,134]. Expanding the results from early studies in the retina [66,77], a recent study reported that capsaicin exposure is sufficient to induce apoptosis in cultured RGCs [72]. Intravitreal injection of capsaicin likewise caused retinal thinning and increased the number of Fluoro-Jade B positive cells in the GCL and INL, particularly in axotomized retinas, an effect that was blocked by capsazepine [94]. Because TRPV1 channels contribute to mechanosensation in some cell types [42,135], but see [136], exposure to hydrostatic pressure was employed to test whether TRPV1 might represent the intrinsic pressure-sensitive pathway through which RGCs respond to elevated IOP. Cultured RGCs and microglia were placed into a pressure chamber and the authors reported increased [Ca^2+^]_i_ when the pressure was raised by 70 mm Hg [71,72]. The observation that excessive TRPV1 activity is excitotoxic for RGCs, potentially due to Ca^2+^ overload [72,137], is a valuable step forward in our understanding of TRPV1 pathophysiology within the retina. By providing evidence that the channel might be intrinsically sensitive to pressure, these data provide an attractive model for mechanically-induced Ca^2+^ dysregulation and RGC remodeling/apoptosis in IOP-induced retinal disease. However, the experimental paradigm has recently been questioned within the context of glaucoma [138], as intended pressure application within the standard laboratory incubator may not have reached the intended pressure differential across the plasma membrane due to non-compressibility of water. Moreover, loss of TRPV1 channels is not protective in mouse glaucoma models [17]. Nonetheless, the mechanobiology of IOP and distribution of mechanical forces across retinal tissue under pathological conditions remains a key challenge in retinal research. This includes characterization of the pressure gradient between the inner and outer eye, which might result in the expansion of the orbit [73] and the optic nerve head [139]. TRPV1, which may sense mechanical stress under certain conditions (such as mechanical hyperalgesia or cell shrinking) [42] might therefore play an accentuated role in retinal pathology.

While TRPV1 activation might be deleterious under some conditions, it has been associated with neuroprotection in other contexts [140]. Recent findings [16], which show that IOP-induced damage to RGCs is augmented in Trpv1^−/−^ animals, suggest that a novel neuroprotective function for TRPV1 channels. RGC injury in Trpv1^−/−^ mice and capsazepine-treated rats was associated with inhibited anterograde axonal transport of a fluorescently tagged marker, axonal loss and astrogliosis in the microbead occlusion glaucoma model. TRPV1 ablation also suppressed a post-IOP exposure-dependent increase in RGC firing and increased the amount of depolarizing current needed to reach the RGC firing threshold. The authors suggest that TRPV1 activity rescues RGCs by promoting their excitability during retinal stress. Another protective mechanism might involve TRPV1-dependent release of the protective interleukin 6 (IL-6) from activated microglia [141], as TRPV1 inhibition by iodo-resiniferatoxin (specific) and Ruthenium Red (non-specific) prevented hydrostatic pressure-induced release of IL-6 and NFκB translocation into the nucleus of microglia [71]. A recent *in vivo* study by Sakamoto *et al.* [142] showed that TRPV1 agonists (capsaicin or SA13353) might evince a paradoxical suppression of NMDA-mediated excitotoxicity of rat RGCs. This effect was prevented by capsazepine, CGRP (8–37) (calcitonin gene-related peptide receptor antagonist) or RP67580 (tachykinin NK_1_ receptor antagonist). CGRP and substance P, which are increased by electrical stimulation of the retina [143] and metabolic stress [144], were protective [142]. Low levels of systemic capsaicin were reported to elevate the CGRP concentration and reduce apoptosis in the GCL of rats with STZ-induced diabetic retinopathy [144]. Taken together, the results from capsaicin exposure studies appear contradictory and unresolved. It appears that TRPV1 activity can be beneficial or detrimental to RGCs depending on the disease model, the extent of TRPV1 activation and activation of auxiliary calcium effectors, however, there is also the serious concern of concomitant overstimulation of capsaicin-sensitive mechanisms such as TRPM1 that needs to be addressed in future work.

### 3.5. TRPV1 and Retinal Vasoregulation

TRPV1 is expressed in vascular endothelial cells and arteriolar smooth muscle cells [20,51,145,146] and may modulate vascular tone and permeability by serving as a source of Ca^2+^ for NO synthesis. Donnerer and Lembeck [147] first showed that capsaicin evokes vascular responses that are independent of sensory innervation. TRPV1’s contribution to the regulation of blood distribution appears to be tissue-specific, as TRPV1 agonists can cause neurogenic vasodilation (in the skin) and vasoconstriction (in skeletal muscle) [148,149,150]. In the retina, TRPV1-ir colocalized with vascular endothelial cells at the inner limiting membrane [92], suggesting that the channel might also modulate vascular flow and/or permeability in this tissue, possibly via Ca^2+^-dependent upregulation of inducible and endothelial NO synthase isoforms [151] and/or production of CGRP [145,152]. eCB analogs reduced proliferation of human retinal vascular endothelial cells [153], suggesting additional roles in the regulation of vascular leakage and integrity of the blood-retina-barrier.

TRPV1 expression in astrocytes lining blood vessels within the inner retina is also likely to modulate glial control of vascular tone and/or Ca^2+^-dependent astrogliosis [101]. Inflammation that accompanies reactive gliosis of protoplasmic astrocytes and Müller glia can itself affect blood-retina-barrier permeability, vascular leakage and oxidative damage [73]. As these events are particularly hazardous to RGCs, both pathological and neuroprotective aspects of TRPV1 and eCB activity may offer potential benefits for treating traumatic, inflammatory and ischemic optic neuropathies in diabetic retinopathy, ischemia, optic neuritis and glaucoma.

## 4. Conclusions

Although TRPV1 expression has been documented across many cell types and in species across phyla ranging from fish to nonhuman primates, at present remarkably little definitive information is available about what the channel is doing in visual processing, retinal development and homeostatic functions of retinal neurons, glia and vasculature. At the very least, it seems that the network comprised of TRPV1 channels, CB1 receptors, eCBs /eicosanoids, kinases and phosphatases (Figure 3) represents a novel neuromodulatory system that could tune the excitability of retinal neurons through amplification or dampening of retinal output and/or contribute to activity-dependent refinement and retinal patterning of visual circuits. The polymodal properties of the TRPV1 channel suggest that, similar to its sibling TRPV4 [17,73], eCB-dependent modulation of Ca^2+^ entry could drive numerous Ca^2+^-dependent processes associated with normal and pathological retinal physiology. The prominent functional expression of TRPV1 channels in RGCs and microglia (Figure 2) and the stupendous complexity of endovanilloid/endocannabinoid signaling (Figure 3) suggest that the channels could modulate the diverse presynaptic inputs to RGCs as well as participate in nonretrograde, light-dependent, modulation of retinal output. It will be important to determine how TRPV1 activators and modulators orchestrate channel properties and its interactions with voltage and calcium effectors such as voltage-operated Ca^2+^ channels, NMDA receptors and Ca^2+^-dependent transcription factors (e.g., NFAT, NFkB, c-fos and DREAM). A particularly interesting area of research will be to evaluate the role of CB1R–TRPV1 interactions, especially under pathological conditions where cannabinoids can provide significant neuroprotection [98]. Given that TRPV1 was identified as a potential intracellular Ca^2+^ release channel [154] studies are also needed to evaluate its relationship to Ca^2+^ release from intracellular stores and activation of store-operated currents, which have been shown to play an increasingly prominent function in retinal Ca^2+^ homeostasis [74,103,155,156]. Because light-dependent changes in [Ca^2+^]_i_ levels are the main driver of tonic neurotransmission that characterizes retinal signaling in light and darkness [157], Ca^2+^ influx and clearance in retinal neurons are under tight control. A clear example of such exquisite control are photoreceptors where µV changes in presynaptic membrane potential translate into discernable differences in [Ca^2+^]_i_ within photoreceptor terminals and visual perception [158,159]. Thus, even modest [Ca^2+^]_i_ elevations mediated by physiological TRPV1 activation could have significant repercussion for downstream visual signaling. Finally, taking into account the reported roles for TRPV1 in hypertonic transduction and mechanosensation [160], it will be of interest whether the channel also contributes to retinal osmoregulation and intrinsic responsiveness to IOP and/or mechanical trauma. This would implicate this polymodal channel in key aspects of retinal homeostasis and response to stress. In summary, the determination of the role that TRP channels play in invertebrate vision together with the elucidation of TRPV1 function in acute and chronic pain represent two of the pinnacles of modern neuroscience. In comparison, TRPV1 research in the mammalian visual system is in its infancy and more studies are needed to better define the involvement of this channel in sensory transduction at the single cell level within the retina, function of visual circuits and the pathogenesis/etiology of blinding diseases.
